# Indoxyl Sulfate, a Gut Microbiota-Derived Metabolite, Modulates Hepatic Cholesterol Metabolism via SREBP-2/HMG-CoA Reductase Upregulation in Rats

**DOI:** 10.3390/nu18132160

**Published:** 2026-07-03

**Authors:** Mateusz Szudzik, Mikołaj Zajdel, Anna Laskowska, Tomasz Hutsch, Marcin Ufnal

**Affiliations:** 1 Laboratory of Centre for Preclinical Research, Department of Experimental Physiology and Pathophysiology, Medical University of Warsaw, Pawińskiego 3c Street, 02-091 Warsaw, Poland; mateusz.szudzik@wum.edu.pl (M.S.); s082807@student.wum.edu.pl (M.Z.); 2 Centre for Preclinical Research and Technology (CePT), Department of Pharmaceutical Microbiology and Bioanalysis, Faculty of Pharmacy, Medical University of Warsaw, 02-097 Warsaw, Poland or anna.laskowska@wum.edu.pl; 3 Department of Pathology and Veterinary Diagnostics, Institute of Veterinary Medicine, Warsaw University of Life Sciences, 02-776 Warsaw, Poland; tomasz_hutsch@sggw.edu.pl

**Keywords:** dyslipidemia, indoxyl sulfate, LDL, bacterial metabolites

## Abstract

Background: A high-fat diet (HFD) contributes to cardiometabolic disease. Gut microbiota-derived metabolites may participate in this process, but their contribution to lipid regulation is not well defined. Indoxyl sulfate (IS), a microbiota-derived metabolite, has been linked to vascular and metabolic dysfunction. Its role in lipid metabolism remains unclear. Methods: In Part A, plasma and urinary concentrations of IS were measured in plasma and urine from HFD-fed rats in which dyslipidemia had developed, together with controls. In Part B, HepG2 cells were exposed to IS, and cell viability and selected cholesterol metabolism-related transcripts and proteins were assessed. In Part C, 10-week-old, male Sprague–Dawley rats maintained on a standard diet received vehicle or IS at two doses for 8 weeks. Hepatic expression of LDLR, SREBP-2, HMG-CoA reductase, and related cholesterol metabolism markers were measured by quantitative real-time PCR and Western blotting. Results: In Part A, higher plasma IS concentrations and higher daily urinary IS excretion were found in samples collected from HFD-fed rats compared to controls. In HepG2 cells, IS reduced cell viability at higher concentrations and increased LDLR mRNA and protein expression. In IS-treated rats, total cholesterol, LDL-cholesterol, and triglycerides increased in a dose-dependent manner. Hepatic SREBP-2 and HMG-CoA reductase protein levels were increased at both IS doses, whereas LDLR protein abundance was increased at the higher dose. Moreover, serum PCSK9 levels were reduced in IS-treated rats. Conclusion: IS increased in HFD-fed rats. IS altered cholesterol metabolism-related pathways in HepG2 cells and in rats. In vivo IS administration increased circulating lipids and hepatic proteins involved in cholesterol synthesis and uptake. These findings indicate that IS may contribute to disturbed lipid homeostasis, although its role in HFD-induced dyslipidemia requires further mechanistic confirmation.

## 1. Introduction

High-fat diets profoundly disrupt lipid homeostasis and promote metabolic and cardiovascular disease. However, the underlying mechanisms remain complex and incompletely understood. We previously demonstrated that a high-fat diet, even under conditions of physiological caloric intake and without inducing obesity, triggers dyslipidemia and MASLD/MASH-like changes and reduces gut microbiota diversity in rats, thereby linking dietary fat intake and gut microbiota to hepatic and systemic metabolic dysfunction [1].

An increasing number of studies demonstrate diet-dependent alterations in gut microbiota composition and the production of microbial metabolites that may mediate systemic metabolic effects [2]. Indoxyl sulfate (IS) is a gut microbiota-derived uremic toxin whose plasma concentrations can reach up to 75 mg/L in advanced chronic kidney disease (CKD) [3]. Indoxyl sulfate (IS) has been shown to promote the uptake of oxidized LDL and impair cholesterol efflux in macrophages [4,5]. However, its effect on lipid homeostasis in vivo and the underlying mechanisms remain incompletely understood. Several studies have suggested that IS may contribute to hepatic dysfunction through the induction of oxidative stress [6], inflammation, [7] and drug metabolism disturbances [8]. However, the direct effects of IS on hepatic cholesterol metabolism in vivo remain poorly characterized. Furthermore, it has not yet been elucidated whether dietary composition influences IS production and thereby contributes to lipid dysregulation. We therefore hypothesized that a high-fat diet may increase IS levels, leading to disruption of lipid homeostasis in rats.

## 2. Materials and Methods

### 2.1. Animals, Cell Culture, and Experimental Design

The experiments adhered to Directive 2010/63 EU. All methods were carried out in accordance with the relevant guidelines and regulations, ensuring compliance with the ethical standards for animal research. All experiments were conducted in accordance with the aforementioned guidelines and regulations, and in particular in accordance with the ARRIVE guidelines. Rats were housed together in polypropylene cages with environmental enrichment, following a 12 h light and 12 h dark cycle. The temperature was maintained at 22–23 °C, with a humidity level of 45–55%. Sprague–Dawley rats were purchased from the Central Laboratory for Experimental Animals, Medical University of Warsaw (Poland). Rats were sacrificed by cervical dislocation at the end of experiments.

Part A

The samples for part A of the present study were obtained from our previous study [1]. The control group received a standard laboratory diet, while the high-fat diet (HFD) group received a diet rich in fats and developed dyslipidemia and dysfunction-associated steatotic liver disease (MASLD). The composition and nutritional value of the diets are presented in Supplementary Table S1. Details of the animal cohorts, dietary interventions, and overall experimental design were described previously [1]. The present study extends previous findings by evaluating serum indoxyl sulfate and demonstrating significant differences between the study groups. These findings provided the rationale for the subsequent experiments de-scribed in Parts B and C, which investigated the effects of IS on lipid metabolism.

Part B

Cell culture

Human hepatoma cells HepG2 (ATCC HB-8065) were cultured in DMEM supplemented with 10% FBS and 1% antibiotic solution (penicillin/streptomycin). For mRNA expression analysis, cells were seeded in 6-well plates at a density of 3 × 10^5^ per well and incubated up to 80% confluence. Next, cells were treated with IS (0.1, 0.25, 0.5, 1, 2.5, 5 mM) for 24 h. Total RNA was isolated using the Total RNA Mini kit (A&A Biotechnology, Gdańsk, Poland) according to the manufacturer’s protocol. The mRNA was subsequently analyzed using RT-qPCR, following the procedure previously described [9]. Quantitative real-time PCR was performed using a Bio-Rad system (Bio-Rad, Hercules, CA, USA). with gene-specific primers for LDLR (qHsaCID0015114) and β-actin (qHsaCED0019162), using iTaq^®^ Universal SYBR Green Supermix. Amplification specificity was confirmed by melting curve analysis and agarose gel electrophoresis. Data were analyzed using CFX Maestro software (Version 4.1.2433.1219) (Bio-Rad, Hercules, CA, USA). Transcript levels were normalized to β-actin, selected using NormFinder (v0.953)

For Western blot cells were seeded in T75 culture flasks at a density of 1 × 10^6^ per flask and incubated up to 80% confluence. Next, cells were treated with IS for 24 h as described above. Then, cells were washed with PBS-EDTA buffer, harvested, and centrifuged. Cells were lysed in RIPA buffer (50 mM Tris-HCl, pH 7.4, 150 mM NaCl, 1% NP-40, 0.5% sodium deoxycholate, 0.1% SDS and 1 mM EDTA) and kept on ice for 30 min, homogenized, and centrifuged. Supernatants were transferred to new tubes, and pellets were discarded. The procedure for Western blot was performed as described for the liver.

Part C

To investigate the impact of IS on cholesterol metabolism, male Sprague–Dawley rats were divided into three groups: controls, 10 IS, and 100 IS (*n* = 6 per group). The randomization was performed using a simple method, specifically by rolling a die to assign animals to experimental groups. They received different doses of IS (10 and 100 mg/kg/day) or an equivalent volume of phosphate buffer saline (controls) through intraperitoneal administration for a duration of 8 weeks. Tissues, plasma, and serum samples from rats were collected in a fasting state (8 h). The ethical approval for this part of the study was granted by the Local Bioethical Committee (no: WAW2/117/2023).

### 2.2. Biochemicals and Metabolites Panel Analysis

Biochemical analysis of plasma (total cholesterol, LDL-C, HDL-C, triglycerides) was performed using a Cobas 6000 analyzer (Roche Diagnostics, Indianapolis, IN, USA).

Plasma and urine concentrations of IS were measured using Waters Acquity ultra performance liquid chromatograph coupled with a Waters TQ-S triple-quadrupole mass spectrometer. The mass spectrometer was operated in multiple reaction monitoring (MRM)–positive electrospray ionization (ESI) mode as previously described [10].

### 2.3. Viability Assay

The viability of the cells was measured after 6, 24, and 48 h of treatment with IS at concentrations of 0.1, 0.25, 0.5, 1, 2.5, 5, 10 mM. At each time point, 20 µL of MTS solution (Promega, Walldorf, Germany) was added to each well and incubated for 2 h. The absorbance was measured at ƛ = 490 nm.

### 2.4. Western Blot Analysis

Liver samples were lysed in histidine–sucrose buffer (30 mM histidine, 250 mM sucrose, 2 mM EDTA, proteases inhibitors, PMSF, pH 7.4). All liver samples were prepared as described previously [9]. Protein concentration was determined using the Bradford Protein Assay (Bio-Rad, Hercules, CA, USA). For all Western blot analyzes, Laemmli sample buffer 4x was added to samples. To determine the levels of SREBP2, LDLR, HMG-CoA reductase, and β-actin, or GAPDH, all samples were resolved by electrophoresis on 10% and 8% SDS/PAGE gels for SREBP2, LDLR, and HMG-CoA reductase, respectively.

Electroblotting of the resolved proteins on PVDF membranes (Bio-rad, Hercules, CA, USA) and their blocking with skimmed milk and incubation with primary (overnight at 8 °C) and secondary antibodies (1 h, room temperature) were performed. Antibody details are given in Supplementary Table S2. For quantitative analysis of protein content, reactive bands were quantified relative to those of β-actin using a ChemiDoc MP Imaging System with Quantity One software (Bio-rad, Hercules, CA, USA, version 4.6.8). For HepG2 cells, the quantification of normalized band intensity was listed below the target protein.

### 2.5. Histopathology of the Liver

Liver tissues were collected from the 3 groups of animals: controls, 10 IS, and 100 IS (*n* = 6 per group). Tissues were fixed in 10% buffered formalin for 48 h, after which they were subjected to the standard paraffin technique, passing the tissues through a series of alcohols, xylene, and to paraffin. The obtained paraffin sections of the liver tissues were stained with standard hematoxylin–eosin staining. Two doctors of veterinary medicine, specializing in laboratory animal pathology, performed the histopathological evaluations. The examination was blinded.

### 2.6. PCSK9 Elisa Test

A rat serum commercial ELISA kit (LS-F22819, LSBio, Seattle, WA, USA) was used according to the manufacturer’s protocol.

### 2.7. Statistical Analysis

The Shapiro–Wilk test was used to assess data normality. Statistical analyses were performed using ANOVA followed by Tukey’s post hoc test or Student’s *t*-test for normally distributed data, and the Kruskal–Wallis or Mann–Whitney test for non-normally distributed data. All analyses were conducted using GraphPad Prism v8.0.1 (GraphPad Software, Boston, MA, USA). A *p*-value ≤ 0.05 was considered statistically significant. The sample size for the high fat diet experiment was calculated for the primary endpoint of a difference in plasma IS concentration between the HFD and controls, with a predicted effect size of 50% difference between groups, assuming a population mean of 3 with an SD of 0.7 (µM). For the IS treatment experiment, the primary endpoint was HMG-CoA with a predicted treatment effect size of 30% in the highest IS dose vs. placebo; α = 0.05 and power = 0.8.

## 3. Results

### 3.1. High Fat Diet Increased Indoxyl Sulfate in Plasma and Urine

Rats on a high fat diet had significantly higher levels of IS in serum (*p* < 0.05), urine (*p* < 0.001), daily urinary excretion (*p* < 0.01) of IS compared to the control group. There were no differences in creatinine clearance between the groups (Table 1).

### 3.2. High-Fat Diet Elevates LDL in Rats

The rats on the high-fat diet exhibited significantly elevated levels of LDL (0.29 ± 0.02 mmol/L (11.32 ± 0.68 mg/dL), *p* < 0.05)) in plasma compared to the control group (0.24 ± 0.01 mmol/L (9.15 ± 0.37 mg/dL)). However, no significant differences were observed between the groups in triglycerides (1.78 ± 0.15 mmol/L (157.50 ± 13.22 mg/dL)) and (1.49 ± 0.12 mmol/L (131.42 ± 10.60 mg/dL)), HDL (1.12 ± 0.05 mmol/L (43.12 ± 1.96 mg/dL)) and (1.23 ± 0.06 mmol/L (47.26 ± 2.16 mg/dL)), and total cholesterol levels (1.52 ± 0.05 mmol/L (58.50 ± 1.93 mg/dL)) and (1.59 ± 0.07 mmol/L (61.14 ± 2.69 mg/dL)) in controls and HFD rats, respectively [1].

### 3.3. Indoxyl Sulfate Affected the Viability of HepG2 Cells and Increased LDLR mRNA and Protein Levels

**Figure 1:**
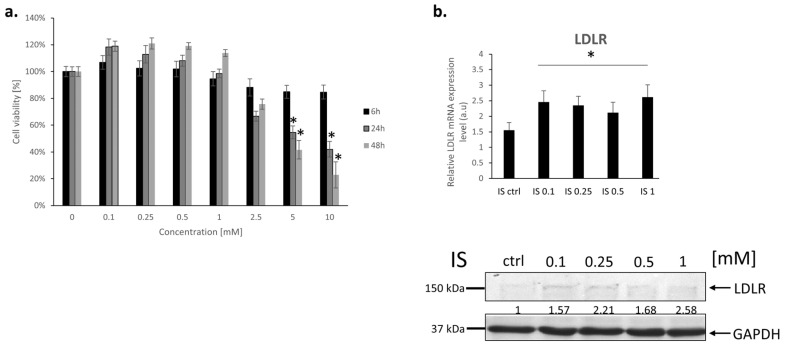
(**a**) MTS viability assay of HepG2 cells treated with indoxyl sulfate at varying concentrations, 0 mM (control), 0.1 mM, 0.25 mM, 0.5 mM, 1 mM, and 5 mM, and 10 mM. Treatments were administered at three time points: 6 h, 24 h, and 48 h.; (**b**) LDLR mRNA and protein expression in HepG2 cells following treatment with indoxyl sulfate at concentrations of 0 mM (control), 0.1 mM, 0.25 mM, 0.5 mM, and 1 mM. Results are normalized to β-actin and expressed as a change relative to their respective controls * *p* < 0.05 vs. control; means ± SE are presented, *n* = 3.

The effect of IS on HepG2 cells was concentration and time-dependent. When treated with concentrations <1 mM IS, the viability of cells was similar to the viability of untreated cells. For IS concentrations >1 mM, a significant decrease in cell viability was observed. This effect was more pronounced after 48 h of treatment (Figure 1a). Therefore, the highest concentrations were excluded from further molecular analyses to avoid the confounding effects of reduced cell viability on the mRNA and protein expression of the investigated gene. IS increased LDLR mRNA and protein in HepG2 cells at all tested concentrations (Figure 1b).

### 3.4. Rats Exposed to Indoxyl Sulfate Develop Dyslipidemia

Rats treated with IS at 100 mg kg^−1^ (body weight) exhibited higher total cholesterol, LDL-cholesterol, and triglycerides than both controls and the 10 mg kg^−1^ IS group (all *p* < 0.05), whereas HDL-cholesterol did not differ between the groups (Table 2).

### 3.5. Indoxyl Sulfate Increased Hepatic LDLR, HMG-CoA Reductase Levels and Decreased Serum PCSK9

**Figure 2:**
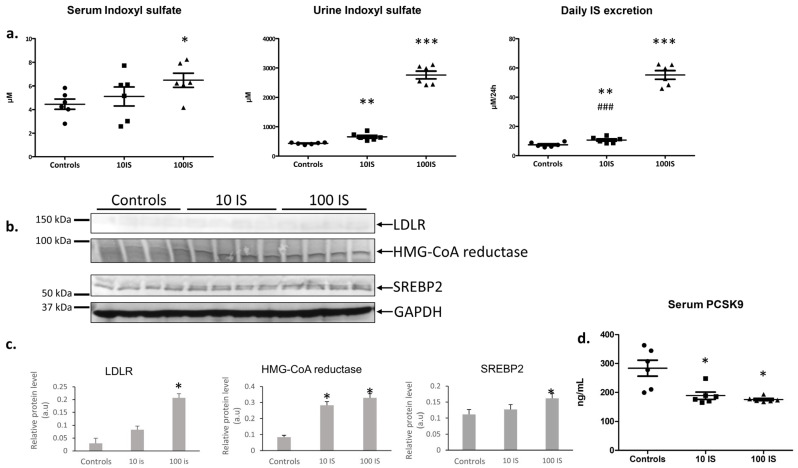
(**a**) Serum and urine IS concentrations, and daily IS excretion in control, 10 IS, and 100 IS rats. (**b**) Representative immunoblots of hepatic LDLR, SREBP2, and HMG-CoA reductase proteins in control, 10 IS, and 100 IS rats. (**c**) Densitometric quantification of each protein relative to β-actin. (**d**) Serum PCSK9 in control, 10 IS, and 100 IS rats. Data are presented as means ± SE for *n* = 6 animals per group.* *p* < 0.05, ** *p* < 0.01,*** *p* < 0.001 vs. controls. ^###^ *p* < 0.05 for 10 IS vs. 100 IS.

In vivo, rats given 100 mg kg^−1^ (body weight) IS had higher serum IS than controls (*p* < 0.05; Figure 2a). Urinary IS was elevated in both the 10 and 100 mg kg^−1^ groups versus controls (*p* < 0.01 and *p* < 0.001, respectively), and daily IS excretion increased in the 10 mg kg^−1^ group versus controls (*p* < 0.01) and further in the 100 mg kg^−1^ group versus both controls and 10 mg kg^−1^ (*p* < 0.001; Figure 2a). At both doses, IS increased SREBP2 and HMG-CoA reductase protein, whereas LDLR protein rose significantly only at 100 mg kg^−1^ (Figure 2b,c). Serum PCSK9 was decreased in both the 10 and 100 mg kg^−1^ groups versus controls (*p* < 0.05), (Figure 2d).

### 3.6. Histopathology of the Liver of IS Treated Rats

**Figure 3:**
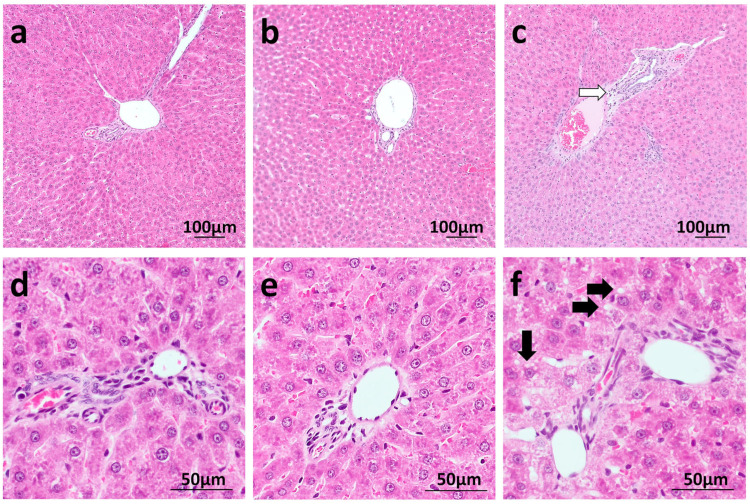
Histopathological image of the liver (**a**,**d**)—control; (**b**,**e**)—dose 10 IS; (**c**,**f**)—dose 100 IS. (**a**–**c**)—lens magnification ×10; (**d**–**f**)—lens magnification ×40. White arrow—focus of edema of the connective tissue in the portal fields; black arrow—vacuolization of hepatocyte cytoplasm, enlargement of hepatocytes and rounding of cell borders; *n* = 6 animals per group.

Histopathological examination revealed no significant pathological changes. However, minimal cytoplasmic vacuolization of hepatocytes was observed in animals receiving 10 mg/kg and 100 mg/kg doses of IS. In the 100 mg/kg group, a slight increase in hepatocyte swelling was noted, accompanied by narrowing of hepatic sinusoidal lumina and mild swelling of connective tissue in the portal areas. (Figure 3).

## 4. Discussion

A key finding was that IS contributes to lipid metabolic dysregulation. Rats fed a high-fat diet showed markedly increased plasma and urinary IS, accompanied by dyslipidemia, without evidence of renal impairment that could explain the increase in plasma IS. In vitro, IS reduced HepG2 cell viability and markedly increased LDLR protein expression. Furthermore, IS-treated rats displayed increased hepatic LDLR and HMG-CoA reductase protein levels. These results indicate that IS may be an active modulator of cholesterol homeostasis. To our knowledge, this is the first study to demonstrate that a high-fat diet increases IS production in rats, linking dietary fat intake to IS-driven metabolic disturbances.

In IS-treated rats, plasma total cholesterol, triglycerides, and LDL-C were increased, accompanied by hepatic induction of HMG-CoA reductase—the rate-limiting enzyme for cholesterol synthesis—suggesting augmented de novo cholesterol production. Hepatic LDLR protein was also elevated, most prominently at higher IS doses. The concordant upregulation of HMG-CoA reductase and LDLR is consistent with activation of a sterol-responsive program; indeed, hepatic SREBP-2, a canonical transactivator of both genes, was increased [11]. Notably, LDL-C rose despite higher total hepatic LDLR, implying that enhanced lipoprotein production and/or impaired receptor function (e.g., post-transcriptional inhibition or trafficking inefficiency) outweighed clearance [12]. Moreover, serum PCSK9 levels were reduced in IS-treated rats, which may partially explain the increased hepatic LDLR protein abundance, given the established role of PCSK9 in LDLR degradation. Nevertheless, the persistence of elevated plasma LDL-C despite increased LDLR suggests that LDL-C homeostasis was influenced by additional mechanisms beyond changes in LDLR abundance alone.

The relationship between lipid metabolism and gut bacteria-derived uremic toxins, such as IS, has not been extensively explored. It has been suggested that IS might contribute to oxidative stress, immune dysfunction, vascular endothelial cell damage, and atherosclerosis among other issues [13]. For instance, Karbowska et al. demonstrated that IS promotes arterial thrombosis in a rat model [14]. Data on the potential contribution of IS to disturbances in lipid homeostasis in vivo are scarce. However, there is some limited research on the impact of IS on cholesterol in vitro. Previous studies suggested that IS may enhance the uptake of oxidized LDL and decrease cholesterol efflux in macrophages [4,5]. The majority of IS research has focused on chronic kidney disease or end-stage renal disease. Plasma IS concentrations reported in CKD rat models may reach levels significantly exceeding those observed in the present study, reaching plasma concentrations of up to 190 µmol/L [15]. In our experiments renal function was preserved, which limited systemic accumulation of IS. Therefore, we propose that elevated IS may contribute to impaired cholesterol homeostasis, particularly in settings associated with increased IS exposure, including HFD, CKD, or the coexistence of both conditions.

In our study, histopathological examination of the liver in rats treated with IS revealed slight hepatocyte swelling, narrowed hepatic sinusoidal lumina, and mild swelling of portal connective tissue, suggesting potential metabolic and/or hemodynamic adaptations to increased liver metabolism without overt pathological changes. In this context, our group previously demonstrated that IS and other indoles affect systemic and liver hemodynamics. These findings may suggest metabolic and/or hemodynamic adaptations of hepatocytes and liver parenchyma, without evidence of overt pathological processes [10,16].

### Limitations and Future Perspectives

This study has several limitations. First, the IS concentrations used in HepG2 cells (Part B) exceeded those measured in HFD rats (Part A), and in vitro exposure may not reflect protein-bound IS handling in vivo. Higher IS concentrations may be relevant to pathological states with impaired renal clearance, such as chronic kidney disease, where circulating IS can rise substantially. However, future studies should test lower, chronic IS exposures, including the 1–10 μM range relevant to HFD-associated IS concentrations. Second, the association between HFD and increased IS remains associative. Gut microbiota composition, microbial indole production, and functional microbiome activity were not directly assessed. Thus, the proposed HFD–microbiota–IS–dyslipidemia axis cannot be established from the present data. Finally, species differences in lipid handling, including lipoprotein distribution and CETP activity, limit direct translation from rats to humans. Further work should include tracer-based cholesterol flux studies, LDLR function assays, microbiome interventions, and cross-species validation.

## 5. Conclusions

In summary, our findings identify IS as a potential modulator of hepatic cholesterol homeostasis. IS was increased after HFD feeding despite preserved renal function, while experimental IS administration increased circulating LDL-C, total cholesterol, and triglycerides in vivo. These changes were accompanied by higher hepatic SREBP-2, HMG-CoA reductase, and LDLR protein abundance, while IS increased LDLR expression and reduced viability in HepG2 cells. These results support a role for IS in disturbed lipid regulation, although its contribution to HFD-induced dyslipidemia requires further mechanistic confirmation.

## Figures and Tables

**Table 1:** * *p* < 0.05, *** *p* < 0.001 vs. controls, means ± SE are presented.

	Controls	HFD	*p*-Value
Serum Indoxyl sulfate (µM)	1.61 ± 0.14	4.01 ± 0.70 *	*p* = 0.013
Urine indoxyl sulfate (µM)	223.35 ± 23.17	466.82 ± 41.66 ***	*p* < 0.001
Daily indoxyl sulfate excretion (µM)	1.099 ± 0.32	1.724 ± 0.50 *	*p* = 0.023
Creatinine clearance (mL/min)	1.15 ±0.12	1.06 ± 0.11	*p* = 0.75

**Table 2:** Lipid profile of IS treated rats.* *p* < 0.05, ** *p* < 0.01,*** *p* < 0.001 vs. controls. ^#^ *p* < 0.05 for 10 IS vs. 100 IS. Means ± SE are presented. (*n* = 6 for each group).

	Controls	10 IS	100 IS	ANOVA
Total cholesterol (mmol/L)	1.698 ± 0.011	1.740 ^#^ ± 0.059	2.067 ** ± 0.104	*p* = 0.003
LDL-C (mmol/L)	0.334 ± 0.004	0.371 ^#^ ± 0.022	0.481 *** ± 0.030	*p* = 0.0008
HDL-C (mmol/L)	1.006 ± 0.019	1.012 ± 0.031	1.116 ± 0.050	*p* = 0.0924
Triglycerides (mmol/L)	0.402 ± 0.044	0.433 ^#^ ± 0.052	0.620 * ± 0.052	*p* = 0.0149

## Data Availability

All data generated in this study are contained within the article or Supplementary Material file.

## Supplementary Materials

The following supporting information can be downloaded at: https://www.mdpi.com/article/10.3390/nu18132160/s1, Table S1: Nutritive value, crude nutrients, and metabolized energy of diets; Table S2: List of antibodies used for Western blot analyses.

## Abbreviations

The following abbreviations are used in this manuscript:

BW	body weight
CKD	chronic kidney disease
HDL	high-density lipoprotein
HepG2	human hepatoma cells
HFD	high fat diet
HMG-CoA reductase	3-hydroxy-3-methyl-glutaryl-coenzyme A reductase
IS	indoxyl sulfate
LDL	low-density lipoprotein
LDLR	low-density lipoprotein receptor
MASH	metabolic dysfunction-associated steatohepatitis
MASLD	metabolic dysfunction-associated steatotic liver disease
SD	Sprague–Dawley
SREBP2	sterol regulatory element-binding protein 2
