# Supplementary material for: Indoxyl Sulfate, a Gut Microbiota-Derived Metabolite, Modulates Hepatic Cholesterol Metabolism via SREBP-2/HMG-CoA Reductase Upregulation in Rats
Source: Nutrients. 2026 Jul 3;18(13):2160. doi: 10.3390/nu18132160 (PMC13364387; doi:10.3390/nu18132160)
Supplement: Supplementary file 1 [file nutrients-18-02160-s001.zip › nutrients-4374540-supplementary.pdf]

Supplemantary Table S1. Nutritive value, crude nutrients and metabolized energy of diets.

	**Control Diet**	**High Fat Diet**
**Metabolized energy [kcal/kg]**	3,514	4,497
**fat [%]**	10	45
**protein [%]**	24	18
**carbonhydrates [%]**	66	37
**Monosaccharides[mg/kg]**	15.143	102.200
**disaccharides[mg/kg]**	117.705	50.355
**polysaccharides[mg/kg]**	427.227	229.252
**moisture [%]**	7.9	3.9
**crude ash [%]**	4.3	3.9
**crude fibre [%]**	3.1	5.6
**crude fat [%]**	4.0	22.6
**crude protein [%]**	20.7	20.8
**Nitrogen free extractives[%]**	60	43.2

Diets used in the study were obtained from Altromin in Lage, Germany.

Supplementary Table S2. List of antibodies used for Western blot analyses

Target protein	Primary Ab	Dilution	Secondary Ab	Dilution
Srebp-2	Rabbit polyclonal, Abcam ab30682	1:1000	Goat anti-rabbit, Abcam ab97048-1	1:10,000
LDLr	Rabbit polyclonal, Abcam ab30532	1:1000	Goat anti-rabbit, Abcam ab97048-1	1:10,000
HMG-CoA reductase	Rabbit polyclonal, Invitrogen, PA5-37367	1:1000	Goat anti-rabbit, Abcam ab97048-1	1:10,000
GAPDH	Goat polyclonal, antibodies.com A83722	1:1000	Donkey anti goat, Abcam ab97107-1	1:10,000
